# Proceedings of the 10th annual deep brain stimulation think tank: Advances in cutting edge technologies, artificial intelligence, neuromodulation, neuroethics, interventional psychiatry, and women in neuromodulation

**DOI:** 10.3389/fnhum.2022.1084782

**Published:** 2023-01-27

**Authors:** Joshua K. Wong, Helen S. Mayberg, Doris D. Wang, R. Mark Richardson, Casey H. Halpern, Lothar Krinke, Mattia Arlotti, Lorenzo Rossi, Alberto Priori, Sara Marceglia, Ro’ee Gilron, James F. Cavanagh, Jack W. Judy, Svjetlana Miocinovic, Annaelle D. Devergnas, Roy V. Sillitoe, Stephanie Cernera, Carina R. Oehrn, Aysegul Gunduz, Wayne K. Goodman, Erika A. Petersen, Helen Bronte-Stewart, Robert S. Raike, Mahsa Malekmohammadi, David Greene, Petra Heiden, Huiling Tan, Jens Volkmann, Valerie Voon, Luming Li, Pankaj Sah, Terry Coyne, Peter A. Silburn, Cynthia S. Kubu, Anna Wexler, Jennifer Chandler, Nicole R. Provenza, Sarah R. Heilbronner, Marta San Luciano, Christopher J. Rozell, Michael D. Fox, Coralie de Hemptinne, Jaimie M. Henderson, Sameer A. Sheth, Michael S. Okun

**Affiliations:** ^1^Department of Neurology, Fixel Institute for Neurological Diseases, University of Florida, Gainesville, FL, United States; ^2^Department of Neurology, Neurosurgery, Psychiatry, and Neuroscience, Icahn School of Medicine at Mount Sinai, New York, NY, United States; ^3^Department of Neurological Surgery, Weill Institute for Neurosciences, University of California, San Francisco, San Francisco, CA, United States; ^4^Department of Neurosurgery, Massachusetts General Hospital and Harvard Medical School, Boston, MA, United States; ^5^Richards Medical Research Laboratories, Department of Neurosurgery, Perelman School of Medicine, Pennsylvania Hospital, University of Pennsylvania, Philadelphia, PA, United States; ^6^Newronika, Goose Creek, SC, United States; ^7^Department of Neuroscience, West Virginia University, Morgantown, WV, United States; ^8^Rune Labs, San Francisco, CA, United States; ^9^Department of Psychology, University of New Mexico, Albuquerque, NM, United States; ^10^Department of Electrical and Computer Engineering, University of Florida, Gainesville, FL, United States; ^11^Department of Neurology, School of Medicine, Emory University, Atlanta, GA, United States; ^12^Department of Neuroscience, Baylor College of Medicine, Houston, TX, United States; ^13^J. Crayton Pruitt Family Department of Biomedical Engineering, University of Florida, Gainesville, FL, United States; ^14^Department of Psychiatry and Behavioral Sciences, Baylor College of Medicine, Houston, TX, United States; ^15^Department of Neurosurgery, University of Arkansas for Medical Sciences, Little Rock, AR, United States; ^16^Department of Neurology and Neurological Sciences, Stanford University School of Medicine, Stanford, CA, United States; ^17^Restorative Therapies Group Implantables, Research, and Core Technology, Medtronic Inc., Minneapolis, MN, United States; ^18^Boston Scientific Neuromodulation Corporation, Valencia, CA, United States; ^19^NeuroPace, Inc., Mountain View, CA, United States; ^20^Department of Stereotactic and Functional Neurosurgery, Faculty of Medicine, University Hospital Cologne, University of Cologne, Cologne, Germany; ^21^Medical Research Council Brain Network Dynamics Unit, Nuffield Department of Clinical Neurosciences, University of Oxford, Oxford, United Kingdom; ^22^Department of Neurology, University of Würzburg, Würzburg, Germany; ^23^Department of Psychiatry, University of Cambridge, Cambridge, United Kingdom; ^24^National Engineering Research Center of Neuromodulation, School of Aerospace Engineering, Tsinghua University, Beijing, China; ^25^Queensland Brain Institute, University of Queensland, St Lucia, QLD, Australia; ^26^Department of Neurology, Cleveland Clinic, Cleveland, OH, United States; ^27^Department of Medical Ethics and Health Policy, University of Pennsylvania, Philadelphia, PA, United States; ^28^Centre for Health Law, Policy, and Ethics, Faculty of Law, University of Ottawa, Ottawa, ON, Canada; ^29^Department of Neurosurgery, Baylor College of Medicine, Houston, TX, United States; ^30^Department of Neuroscience, University of Minnesota, Minneapolis, MN, United States; ^31^Department of Neurology, Weill Institute for Neurosciences, University of California, San Francisco, San Francisco, CA, United States; ^32^School of Electrical and Computer Engineering, Georgia Institute of Technology, Atlanta, GA, United States; ^33^Center for Brain Circuit Therapeutics, Department of Neurology, Psychiatry, Radiology, and Neurosurgery, Brigham and Women’s Hospital, Boston, MA, United States; ^34^Department of Neurosurgery, Stanford University, Stanford, CA, United States

**Keywords:** deep brain stimulation (DBS), artificial intelligence, neuroethics, Parkinson’s disease, dystonia, interventional psychiatry, adaptive DBS, epilepsy

## Abstract

The deep brain stimulation (DBS) Think Tank X was held on August 17–19, 2022 in Orlando FL. The session organizers and moderators were all women with the theme *women in neuromodulation*. Dr. Helen Mayberg from Mt. Sinai, NY was the keynote speaker. She discussed milestones and her experiences in developing depression DBS. The DBS Think Tank was founded in 2012 and provides an open platform where clinicians, engineers and researchers (from industry and academia) can freely discuss current and emerging DBS technologies as well as the logistical and ethical issues facing the field. The consensus among the DBS Think Tank X speakers was that DBS has continued to expand in scope however several indications have reached the “trough of disillusionment.” DBS for depression was considered as “re-emerging” and approaching a slope of enlightenment. DBS for depression will soon re-enter clinical trials. The group estimated that globally more than 244,000 DBS devices have been implanted for neurological and neuropsychiatric disorders. This year’s meeting was focused on advances in the following areas: neuromodulation in Europe, Asia, and Australia; cutting-edge technologies, closed loop DBS, DBS tele-health, neuroethics, lesion therapy, interventional psychiatry, and adaptive DBS.

## Introduction

**FIGURE 1:**
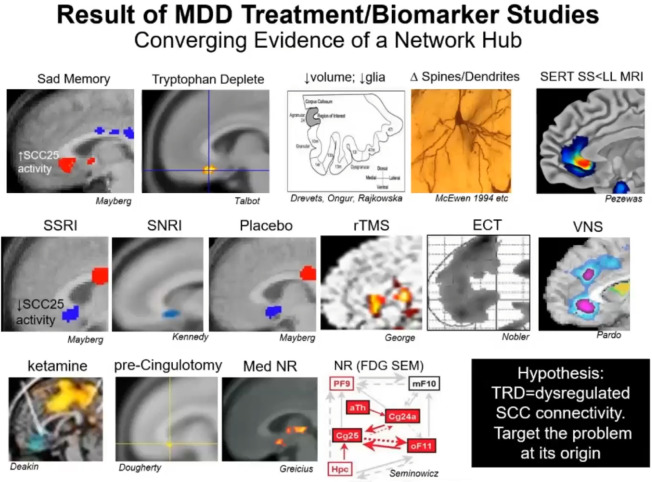
Understanding where sadness is–This figure illustrates various neuroanatomical localizations of key nodes related to depression as a psychological state, the pharmacological treatment of depression, neuromodulation of depression, and recovery from depression. Through a series of small experiments, this collective information has built a network that identified connectivity to the SCC as a critical player in treatment resistant depression. MDD, major depressive disorder; TRD, treatment resistant depression; SCC, subcallosal cingulate.

The 10th annual DBS Think Tank had a theme of *women leaders in neuromodulation* and Dr. Helen Mayberg was the invited keynote speaker. In her talk, Dr. Mayberg emphasized that the DBS field needs hedgehogs, foxes, and chimera. Hedgehogs are useful because of their deep knowledge. This knowledge is more than knowing the rules and cases. It is knowing how all the pieces fit together. Foxes are useful, because they have perspective. Foxes can see the forest and how the trees fit together. Finally, chimera are useful because they can bring together hedgehogs and foxes into effective teams. She emphasized that her mentor taught her to find a problem you care about and to focus on studying a disease and not a method. Her mentor stressed that “methods change.” Dr. Mayberg illustrated how hedgehogs, foxes and chimera need to interact with each other in the pursuit of science using her own experiences over the past three decades asking questions about sadness and depression. Starting with sadness, she and her team developed imaging, biomarkers and treatments, all based on understanding the underpinnings for the network underlying depression (Figure 1). This work led to a series of pilot studies which contributed to refinement of the DBS target for depression. Her studies informed the reasons for the shortcomings of the recent industry based clinical trials of DBS for depression. Collectively, the science has driven a refined approach for depression DBS, which will soon re-enter large scale clinical trials. As there are several budding and emerging disciplines within the field of DBS, Dr. Mayberg advises that this team-based approach with core role players is key to successfully exploring the science.

## Bench therapies inspiring neuromodulation

Neuromodulation therapies have been largely developed using a human intra-operative and post-operative learning approach. In the last decade, however, there has been an explosion in bench neuromodulation-based research. Terms like “optogenetically inspired DBS” have recently emerged and there has been a greater focus on “mechanism of action” and development of animal models of DBS (Vedam-Mai et al., 2021). Animal models of DBS have driven a re-birth of a variety of DBS targets such as cerebellar DBS.

### Optimizing DBS using physiologic signals and biophysical modeling

Programming optimization remains a critical clinical challenge that continues to hamper efficient and widespread use of deep brain stimulation (DBS) therapy. Automated programming using closed-loop paradigms has been an exciting and important next step in the development of next generation DBS therapy. Control signals for using with automated programming strategies can be obtained from kinematic (e.g., accelerometer, gyroscope), electrophysiologic (e.g., evoked potentials, beta power), and/or imaging (e.g., patient-specific activation models) domains. These strategies can provide quantifiable metrics for disease or symptom severity, treatment efficacy, target engagement, and side effect severity. Automated programming for tremor suppression in patients with Parkinson’s disease (PD) and essential tremor has also been successfully implemented and piloted using kinematic signals (Haddock et al., 2018; Sarikhani et al., 2022). Bayesian optimization with safety constraints has further enabled safe and efficient programming with comparable tremor outcomes when compared to programming that is performed by expert clinicians (Sarikhani et al., 2022). Image-guided programming aims to use patient-specific computational DBS activation models to choose stimulation settings in order to maximize computationally predicted stimulation effects in the target region and to avoid side-effects. Computational DBS activation models should be systematically validated to assure that predictions are sufficiently accurate to be reliably useful and applicable in clinical practice. This outcome can be potentially accomplished by comparing *in vivo* electrophysiologic measures of pathway activations with model predictions from the same set of subjects (Miocinovic et al., 2018; Howell et al., 2021). Model performance is critically dependent on accurate lead localization and appropriate selection of pathway excitability. Model accuracy will perform better for omnidirectional rather than directional settings. Further model development will help to resolve these issues for the field (Howell et al., 2021).

### Contribution of non-human primate model to DBS therapy

Non-human primate (NHP) models can be a useful tool in the discovery and improvement of DBS therapy. DBS can possibly reduce the frequency and severity of seizures, however few patients convert to seizure freedom. The NHP model of penicillin-induced seizures is an “on-demand model” for focal seizures (cortical and temporal lobe) that can be used to characterize the anatomically relevant pathways involved in seizure propagation and to identify critical nodes for DBS intervention. Dr. Devergnas used this model to study the involvement of the basal ganglia in the control of cortical seizures and observed a significant but moderate seizure reduction with subthalamic nucleus DBS. Dr. Devergnas then focused her lab’s work on the thalamo-cortical loop and observed strong entrainment of the thalamic cells suggesting that modulation of this specific network might be helpful in desynchronizing cortical activity. Additionally, this model may manifest comorbidities similar to human patients. They recently validated the use of this NHP penicillin model to study the comorbid sleep disorder associated with seizures. Among the different nuclei implicated in sleep activity, they will now investigate the impact of seizures on the pedunculopontine nucleus and the lateral hypothalamus. Activity in both of these regions has shown to be related to control arousal and to regulation of rapid eye movement. Despite not being a classical epilepsy model, the NHP model of penicillin-induced seizures can help us to better understand the pathological mechanisms of seizures and to facilitate the development of new DBS therapies and technologies.

### Cerebellar deep brain stimulation

The cerebellum is well-known for its important roles in motor behaviors including coordination, learning, and posture. However, recent work has revealed its involvement in cognitive behaviors such as language, emotion and reward. Consistent with its diverse behavioral influence, cerebellar function is disrupted in ataxia, tremor and dystonia, as well as in autism spectrum disorders, schizophrenia, and obsessive-compulsive disorder. In a series of recent studies, Dr. Sillitoe and colleagues focused their attention on cerebellar motor function in order to test how a single brain region could contribute to such a wide variety of disorders (van der Heijden et al., 2021; Brown et al., 2022; Liang et al., 2022; Liu et al., 2022). They tested two hypotheses. First, they tested whether specific cerebellar connections may have a more predominant deficit in one disease versus another. Second, they tested whether distinct, abnormal neural signals could be produced in different cerebellar diseases. Using genetic manipulations and *in vivo* electrophysiology, the Sillitoe lab found evidence to support both hypotheses. They found that synaptic contacts onto Purkinje cells are differently, but not exclusively involved in ataxia, tremor and dystonia, and depending on the disease, distinct patterns of Purkinje cell to cerebellar nuclei miscommunication are initiated (van der Heijden et al., 2021). These results motivated them to test whether DBS could be targeted into the cerebellum as a means to compensate for or correct circuit defects and to potentially restore motion. They found that DBS directed to the interposed cerebellar nuclei, which are critical for ongoing motor functions, corrects movement in a range of motor conditions. These data raise the intriguing possibility of extending cerebellar DBS for use in neuropsychiatric conditions.

## Advances and challenges in applying closed loop physiology to neuromodulation

The notion that a device-based approach could be used to decode symptoms and neurophysiology underpinning specific bothersome symptoms has provided excitement which has been driving the development of “closed-loop” or adaptive DBS. In practice, however, there have been formidable challenges as well as opportunities which will all need to be addressed in order to advance a practical and deployable approach.

### At-home adaptive deep brain stimulation for Parkinson’s disease using individualized neural biomarkers

Patients with PD can experience residual motor fluctuations during optimized continuous deep brain stimulation (cDBS). Previous in-clinic and short at-home studies have suggested that adaptive DBS (aDBS), i.e., titrating stimulation amplitude in response to symptom-state-associated neural signals (i.e., biomarkers), could possibly alleviate residual symptoms (Little et al., 2013; Arlotti et al., 2018; Gilron et al., 2021a). It has been uncertain if these results can be replicated at-home for sustained periods. Here, Dr. Cernera and Dr. Oehrn present a pipeline for developing at-home aDBS based on long-term intracranial recordings derived from the subthalamic nucleus (STN) and sensorimotor cortex of five PD patients implanted with the Medtronic Summit*™* RC + S system. Biomarker identification during stimulation was challenging, as stimulation can suppress or enhance frequency-specific neural activity and motor fluctuations during cDBS and these were not associated with STN beta oscillations (13–30 Hz) in all patients (Little et al., 2013). Therefore, it was necessary to explore the whole power spectrum beyond the beta frequency band and, if available, in multiple brain sites. Thereafter, aDBS parameters were selected in a data-driven manner and optimized parameters based on clinical effects during short-term testing (24 h). Using this pipeline, the first long-term at-home randomized double-blind comparison between aDBS and cDBS in one patient (4 weeks per condition in week-long blocks) using an individualized off-state biomarker (∼12 Hz STN) revealed that aDBS increased on-time. Single-blind randomized comparisons between aDBS (8 days) and cDBS (5 days) in another patient using 65 Hz precentral cortical power as an on-state biomarker demonstrated that aDBS reduced dyskinesia and bradykinesia. These lessons could be possibly extended to other indications and could provide key insights for the development of at-home aDBS algorithms.

### Closed-loop deep brain stimulation for Tourette syndrome

Tourette syndrome (TS) is a continuous lifelong syndrome that can be debilitating and stigmatizing for patients with moderate to severe motor and vocal tics that are resistant to medication and to behavioral intervention (Jankovic, 2001; McNaught and Mink, 2011). DBS has emerged as a promising treatment option for addressing medication resistant tics (Ackermans et al., 2011). Over the last few years, Dr. Gunduz and colleagues have demonstrated several key findings that provide the necessary foundation for a prospective trial to test closed-loop neuromodulation for tic suppression. They demonstrated that TS DBS could be successful even if not administered chronically and continuously (Okun et al., 2013; Rossi et al., 2016) which paved the way for closed-loop DBS. A follow-up study examined the thalamic activity in relation to tics recorded from contacts on the DBS lead (Molina et al., 2018). This study uncovered an electrical signal correlating both with occurrence of tic and with clinical improvement. Most recently, they reported thalamo-cortical network characteristics underlying tic generation through the use of deep DBS leads, along with chronically implanted subdural strips (Cagle et al., 2020). The technique was able to separate voluntary movement from tics and demonstrated that DBS could drive brain activity to a healthy, tic free state in the thalamus. These features allowed us to develop embedded closed-loop DBS for TS and to show its feasibility, safety and possible effectiveness when compared to conventional TS DBS for the treatment of tics (Cagle et al., 2022).

### aDBS for intractable OCD: Progress and challenges

Ventral striatum (VS) DBS holds a FDA Humanitarian Device Exemption (HDE) approval for treatment of severe and intractable OCD. Although VS DBS reveals benefit in about 50–66% of cases, there is room for improvement in both clinical benefits and in reduction of DBS-induced behavioral side effects, especially hypomania (Goodman et al., 2021). Dr. Goodman and colleagues reported the preliminary findings from an NIH-funded study to develop adaptive DBS (aDBS) using devices that can both stimulate and record (McLaughlin et al., 2021; Provenza et al., 2021). The study was conducted with the objective of identifying the neural based classifiers for OCD-related distress and DBS-induced hypomania. Building LFP based classifiers for psychiatric states can be challenging because most of our measures are subjective and not on same time scale as the neural recordings. Using computer vision machine learning approaches has been useful as a label for changing mood states. A combination of symptom provocations (e.g., tasks, exposure/response prevention therapy, and naturally occurring exacerbations) can be used to capture changes in OCD symptom severity. In the clinic setting, they use DBS induced mirth response and talkativeness as a proxy for hypomania. The Goodman lab is currently processing and analyzing LFP data time-locked with DBS, tasks, and activity during different behavioral states in the clinic setting and at home, in order to identify neural based classifiers associated with these states.

## Transforming the OR into the laboratory

One shortcoming of neuromodulation research has been the ability of animal models to recapitulate the nuances of the human condition. Several laboratories have adapted the intra-operative environment to become a true laboratory. This approach is useful in understanding movement, speech, appetite or other human behaviors.

### Understanding movement control during DBS surgery

**FIGURE 2:**
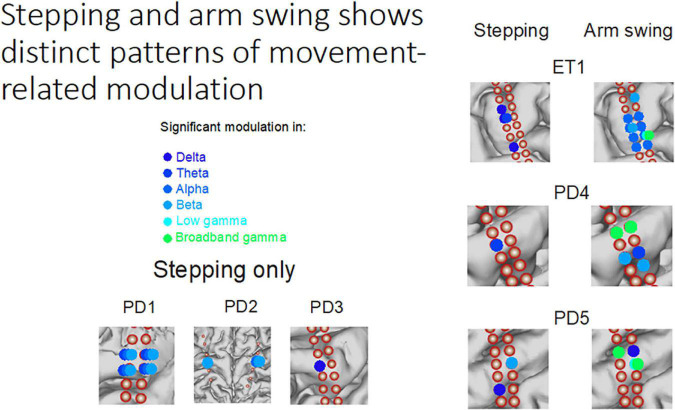
Stepping and arm swing shows distinct patterns of movement-related modulation–Subdural electrocorticography recordings demonstrate significant modulation of various frequency bands while performing different movement tasks. The modulation is unique from both a neuroanatomical perspective and an electrophysiologic perspective.

Awake DBS surgery provides a unique opportunity to learn about movement control. Human bipedal walking involves the complex coordination of leg and arm swing between two sides of the body. How the primary motor cortex coordinates these precisely timed upper and lower extremity movements during locomotion is unknown. Dr. Wang’s intraoperative team recorded subdural electrocorticography activities from the hand/arm area in the primary motor cortex of subjects undergoing DBS surgery who performed stepping and arm swing tasks (Figure 2). They showed that there were stepping-related low frequency oscillations over the arm area (Louie et al., 2022). Furthermore, they found that this oscillatory activity was separable, both in frequency and spatial domains, from gamma band activity changes occurring during arm swing (Louie et al., 2022). This low frequency activity during stepping could serve to entrain and to synchronize upper limb movements during walking. These findings broaden our understanding of motor cortical activity during gait and suggest a potential mechanism for coordinating multiple limb movements during bipedal walking.

### Studying speech production during DBS implantation

**FIGURE 3:**
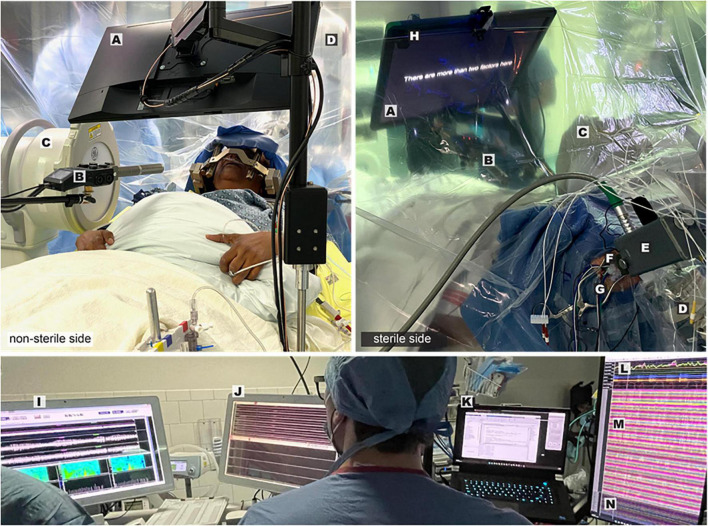
Intraoperative speech production research employing simultaneous cortical and subcortical recording–Views from the non-sterile (left panel) and sterile (right panel) sides of the operative drape show important components of the experimental and clinical set-up: Presentation monitor displaying a sentence stimulus **(A)**, boom microphone and recorder **(B)**, fluoroscopy machine **(C)**, robotic stereotactic arm **(D)**, microdrive **(E)**, tissue glue-filled 14 mm burr hole **(F)**, exiting wires from ECoG electrode strips **(G)**, photodiode **(H)**. Visualization of multiple synchronized recording streams is seen in the lower panel: clinical microelectrode signals **(I)**, macroelectrode signals and task triggers **(J)**, task control/data storage computer **(K)**, audio and respiratory signals **(L)**, ECoG signals **(M)**, DBS lead channels **(N)**.

Speech relies on basal ganglia-thalamocortical network activity, however ideas about how the basal ganglia modulates speech are primarily theoretical. The recent development of experimental paradigms to simultaneously record electrocorticography (ECoG) and subcortical activity during speech in patients undergoing DBS surgery, however, is providing insights into motor speech information coding within these circuits (Figure 3). It is important to note that research related ECoG collected during DBS surgery confers no defined risk to safety or accuracy (Panov et al., 2017; Sisterson et al., 2021). Initial speech studies focused on the STN, where microelectrode recordings revealed the presence of separate populations of neurons whose firing rates selectively decreased during speech planning or increase during speech production (Lipski et al., 2018). Consistent with a role in movement gain, STN gamma activity tracked with specific articulatory motor features, while the strength of theta/alpha oscillatory activity was associated with vocal gain adjustment (Chrabaszcz et al., 2019; Dastolfo-Hromack et al., 2022). In addition, the effort required to produce novel words was reflected in increased gamma activity in both the STN and thalamus (Chrabaszcz et al., 2021; Wang et al., 2022). Methods previously established for antidromic mapping of the human hyperdirect pathway, using cortical potentials elicited from STN stimulation, provided evidence for monosynaptic connections from opercular speech cortex to the STN, including auditory cortex (Miocinovic et al., 2018; Chen W. et al., 2020; Jorge et al., 2022). These findings establish a basis for continued investigation of subcortical participation in speech planning and modulation, including the integration of information from sensory cortical areas participating in both feed-forward and feedback processes. Important additional factors for the future of this field will be the detection of speech artifacts in gamma frequencies and the need to engage patients proactively in intracranial human neuroscience experiments (Montreuil et al., 2019; Bush et al., 2022).

### Provoking human nucleus accumbens representations of appetition

**FIGURE 4:**
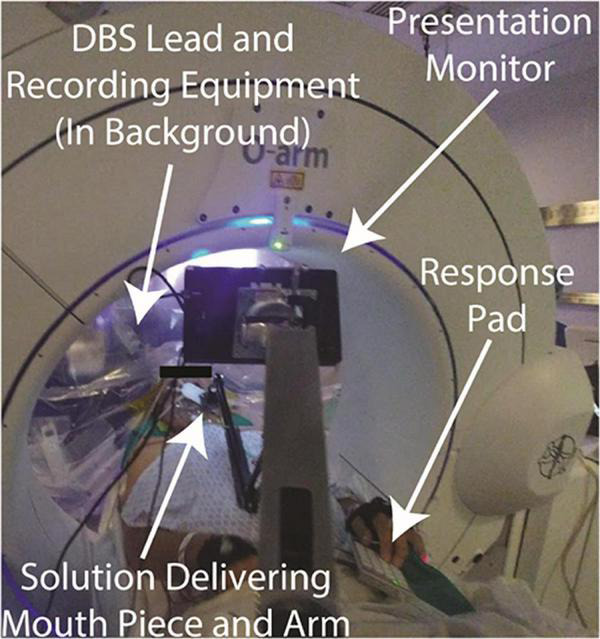
Intraoperative appetition provocation set-up.

Dysregulation of mesolimbic circuits has been implicated in psychiatric disorders as well as in obesity. Increased power in low frequency oscillations have been reported to predominate in the mouse nucleus accumbens (NAc) during moments of heightened appetition (Wu et al., 2018). These field potentials were recorded specifically from the NAc shell subregion and exhibited significant spike-field coherence and correlated with an increased spike rate. Moreover, when used as a biomarker for responsive DBS, this low frequency domain effectively triggered brief bouts of DBS and reduced binge-eating behavior in mice. In an attempt to isolate these appetitive units within this NAc subregion in an ongoing first-in-human trial of responsive DBS for obesity (NCT03868670), tractographic segmentation of the human NAc was used and revealed a ventral posteromedial cluster, demarcating the homologous shell subregion (Cartmell et al., 2019). This territory and its prefrontal interconnections exhibited perturbed structural and functional connectivity in binge-prone obese patients, further implicating disease-specific dysregulation to be mapped during DBS (Barbosa et al., 2022). The Halpern lab used a protocol developed to provoke appetition as illustrated in Figure 4 (Miller et al., 2019). Thus, physiologic representations of appetition within the human NAc defined by tractography may further inform spatial topography of this key mesolimbic node and may confirm patient-specific circuit engagement.

## Cutting edge technologies from the industry sector

A key aspect to the success of the DBS Think Tank is the understanding that it will take industry and academic collaboration in order to advance neuromodulatory therapies into clinical practice. Each year we have an industry blitz to explore cutting edge therapies, challenges and opportunities in transforming science into product.

Innovation in DBS is dependent on cooperation between researchers and industry partners. In order to keep pace with discoveries in the research lab, industry must continue on a trajectory of progress that includes software and hardware innovation, and a trajectory that can incorporate new outcome measures, neurophysiological and biometric information, and the lessons gleaned from “big data.” Highlights from industry revealed the development of new platforms to manage the increasing volume of data generated from patient-implanted devices and how the appropriate use of these data can lead to optimization of DBS with better power consumption and improved patient outcomes. Key concerns in this era of innovation include research platform access as well as data privacy and security. An open channel of discussion between researchers and industry engineers should be maintained, so that clinical research platforms and specialized settings can be accessed outside of commercial applications in order to facilitate experimentation. As data sets become larger and closed loop stimulation exits the laboratory setting and is accessible for the patient at home, security and privacy of data remain essential. Several manufacturers have already encountered novel questions about access to data, and these logistical and ethical issues must be part of the larger conversation on the future of data use.

### Utilizing BrainSense technology to guide DBS therapy

In 2020, the Medtronic Percept*™* PC DBS device with BrainSense*™* technology received US FDA approval and EU CE Mark (Paff et al., 2020; Goyal et al., 2021; Jimenez-Shahed, 2021). The device is capable of delivering electrical stimulation therapy while recording local field potentials (LFP) through the same DBS lead. Since approval, more than 18,000 Percept*™* PCs have been implanted, uniquely enabling the chronic monitoring of brain activity in DBS patients during routine clinical care. The Percept*™* PC hardware platform is also upgradeable through software and firmware unlocks for performance enhancing and clinically meaningful updates.

The commercial availability of chronic LFP sensing also offers the potential to accelerate the pace of translational DBS research. Whereas Medtronic’s first and second-generation DBS + sensing systems, Activa*™* PC + S and Summit*™* RC + S, have been utilized in dozens of investigational neurology studies, access to the technology was limited. In contrast, Percept*™* PC is now approved in the worldwide market for treating on-label indications including Parkinson’s disease (PD), essential tremor (ET), dystonia, obsessive compulsive disorder (OCD), and epilepsy.

Unsurprisingly, brain sensing research has accelerated following Percept*™* PC approval. Initial studies in PD demonstrated that the LFP beta power strength (e.g., 13–30 Hz) correlates with patient akinetic rigidity symptoms and the responses to DBS and medication therapies (Feldmann et al., 2021, 2022; Koeglsperger et al., 2021); key replications of prior studies using investigational recording configurations (Neumann et al., 2016; Ozturk et al., 2020). Moreover, studies across multiple centers demonstrate the capability of Percept*™* PC to routinely detect LFP signals of interest in PD, ET, dystonia, OCD and epilepsy subjects with standard of care procedures (Fasano et al., 2021; Goyal et al., 2021; Thenaisie et al., 2021; Buijink et al., 2022; Vissani et al., 2022), which is also consistent with prior studies using investigational recording devices (Case et al., 2020; Darcy et al., 2022). Further, there is a growing body of case studies demonstrating the application of brain sensing for personalized treatment including initial DBS programming (Fasano et al., 2021; Sirica et al., 2021), DBS and medication optimization (Kern et al., 2022), and even understanding circadian patterns (van Rheede et al., 2022).

In parallel, there are nearly a dozen ongoing trials evaluating the safety and effectiveness of LFP-beta controlled adaptive DBS (aDBS) in PD using the Summit*™* RC + S or the Percept*™* PC aDBS unlock. In the US, EU and Canada the ADAPT PD approval trial (NCT04547712) evaluating aDBS has completed enrollment. In Japan, where aDBS is approved, early case study results are promising (Nakajima et al., 2021; Kimura et al., 2022; van Rheede et al., 2022) and continue to build upon the evidence for patient benefit suggested by previous investigational pilots (Little et al., 2013; Arlotti et al., 2018; Velisar et al., 2019). Finally, several physician-sponsored NIH BRAIN Initiative trials are applying the Summit*™* RC + S to investigate new indications including treatment resistant depression, Tourette’s syndrome and opioid use disorder. Although the early experience with aDBS has been promising, experts at the DBS Think Tank discussed the battery drain issues associated with chronic sensing and the need to address these issues with rechargeables and potentially other technologies. Overall, this broad access to commercial DBS devices with embedded sensing technology has significantly impacted the journey toward personalized care strategies in established indications and biomarker discovery in new indications.

### Toward personalized deep brain stimulation

Boston Scientific Neuromodulation (BSN) focuses on using data, algorithms, and stimulation technologies to personalize therapy for every patient through the following modalities:

*Making imaging available during live programming, effectively aggregating prior neuroimaging and clinical data to aid in titration and leveraging analytics for faster DBS workflows.* Platform supporting this vision, developed in collaboration with Brainlab Inc., include commercially available GUIDE XT and STIMVIEW XT which integrate BSN’s Stimulation Field Models (SFMs) with automatic detection of lead location and orientation, and places these models in an auto-segmented, patient specific anatomy (Lange et al., 2021; Waldthaler et al., 2021). More recent advances include the DBS Illumina 3D algorithm which is an optimization algorithm that synthesizes imaging-based information to optimize SFM size, shape, and location to accelerate Image Guided Programming (Malekmohammadi et al., 2022). BSN is working to make this algorithm commercially available in the future.

*Incorporating objective clinical measurements provided by external sensors into DBS programming using the DBS CLOVER search routine.* CLOVER suggests a stimulation setting, and after testing and assessing the clinical response, including using objective-measure wearables, the algorithm suggests next settings. The search continues until the improvement target for the assessed clinical response has been reached or the search space has been fully explored.

The first version of CLOVER delivered significant reduction of clinical symptoms in few steps using a single symptom; however, patients have clinical profiles including multiple symptoms (Sasaki et al., 2021; Wenzel et al., 2021). The new study with CLOVER uses a multi-symptom, weighted Patient Specific Metric as the input which synthetizes the global clinical state of the patient. The algorithm significantly reduces the UPDRS PIII scores (comparable to standard of care), and in fewer programming steps. BSN is working to make this algorithm commercially available in the future.

*Enabling research on novel stim patterns to effectively explore the time domain aspects of DBS.* Over the past decade, there has been increased interest in exploring temporal and spatial variations of DBS. To enable and accelerate research in this field BSN has released a new research software called Chronos (Wong et al., 2022). Chronos utilizes stimulation capability existant in Boston Scientific’s rechargeable Genus stimulators to enable stimulation over an expanded range of frequencies and pulse-widths, bursting and cycling of stimulation on and off over multiple timescales, and generation of spatio-temporally complex patterns pulse-by-pulse. All stimulation delivered using Chronos enforces commercial charge density and amplitude safety limits and can be controlled using the patient’s existing Remote Control. Chronos is intended for investigational use in selected studies, contingent on required approvals from Investigational Review Boards and, if required, IDE approval from the FDA.

### Updates in neuromodulation for epilepsy

NeuroPace presently has three active clinical trials studying neuromodulation in epilepsy. The RESPONSE Study (ClinicalTrials.gov: NCT04839601) is a pivotal study to determine whether the RNS System is safe and effective as an adjunctive therapy in individuals ages 12 through 17 years who have drug-resistant focal epilepsy.

The NAUTILUS Study (ClinicalTrials.gov: NCT05147571) is a pivotal study to determine if the RNS System is safe and effective in individuals 12 years and older who have drug-resistant idiopathic generalized epilepsy (IGE). The study is a prospective, multicenter, single-blind, randomized, sham stimulation controlled pivotal study that will enroll 100 participants within the United States. Patients must have a confirmed diagnosis of IGE consistent with the ILAE Revised Classification of Seizures experiencing generalized tonic-clonic seizures (GTC), with or without myoclonic or absence seizures (Fisher et al., 2017). Leads will be placed bilaterally in the centromedian nuclei. Primary outcome measures are the 12-week post-operative serious device-related adverse event rate, and the time to second GTC seizure.

The RNS System Lennox-Gastaut Syndrome (LGS) Feasibility Study (ClinicalTrials.gov: NCT05339126) is an NINDS funded Brain Initiative study. The study is intended to generate preliminary safety and effectiveness data for brain-responsive neurostimulation of thalamocortical networks as an adjunctive therapy to reduce generalized seizures in individuals 12 years of age or older with LGS who are refractory to antiseizure medications. Up to 20 subjects will be enrolled. Pre-operative imaging will be used to create patient-specific maps of seizure networks, providing insight into how to personalize the treatment for each participant. Leads will be placed bilaterally in pre-frontal cortex and centromedian nuclei.

## AlphaDBS is a new implantable closed-loop clinical neural interface

The AlphaDBS system features advanced filtering technology for detecting local field potentials (LFPs) sensed through the DBS lead (Arlotti et al., 2021; Marceglia et al., 2022). The implanted stimulator also utilizes a linear control algorithm that adjusts stimulation parameters according to the power in a selectable frequency band of LFPs. The system has 16 independently controlled stimulation and two sensing channels, one per hemisphere. *Via* a telemetry unit and a patient app, LFP data recorded 24/7 can be uploaded to a cloud-based database, with no data loss or overwriting. The fully implantable system has received CE-Mark for conventional DBS for the treatment of PD but not for adaptive DBS. The system is not FDA approved.

**FIGURE 5:**
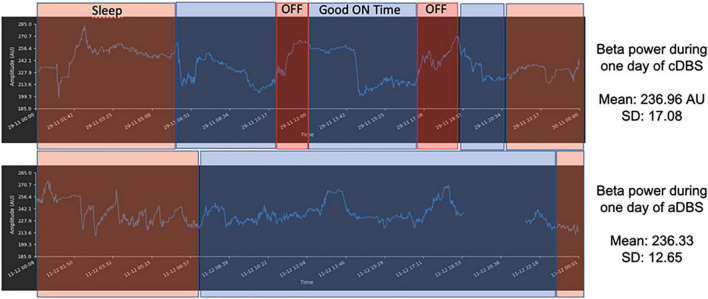
Example of 1 day recording with matched diary features–Sleep–orange rectangles; OFF time–red rectangles; ON time without troublesome dyskinesias (Good on Time)–blue rectangles for AlphaDBS system in cDBS **(top row)** and aDBS **(bottom row)**. The x-axis is time (sampling frequency: 1 min) and the y-axis is beta power amplitude (arbitrary units–AU). The signal displayed was stored within the AlphaDBS IPG with the patient at home and was uploaded to the cloud system *via* telemetry and patient app, according to the data management protocol.

An external version of the system was tested in several recently published clinical trials in advanced PD patients with encouraging results suggesting that adaptive DBS improves clinical outcomes, specifically reducing dyskinesias. The fully implanted system has been tested in an ongoing pilot study in PD patients requiring an implantable pulse generator exchange utilizing the quadripolar leads already implanted in the STN. Six patients have been implanted to date. The system is characterized by reliable artifact-free recording and distributed neural data and signal management protocols (Figure 5). Alpha DBS’s present application in the ongoing study represents a “proof of functioning” of a clinically viable implanted brain-computer interface (BCI) for adaptive DBS.

### Real-world monitoring data can inform Parkinson’s management

**FIGURE 6:**
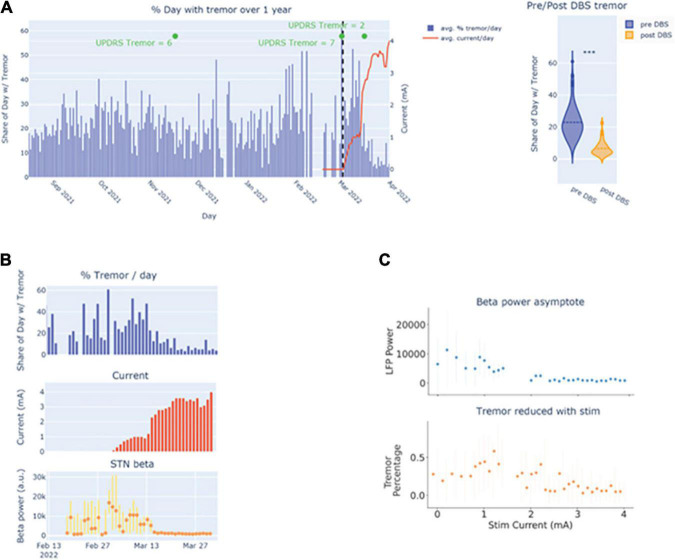
**(A)** Visualization of tremor burden over time. **(B)** Viewing the temporal relationship between tremor burden with stimulation amplitude and beta power. **(C)** Visualizing the relationship between tremor and beta power with stimulation amplitude.

Basal ganglia LFP sensing, which is currently embedded in commercially available DBS devices, provides a rich dataset that may aid the development of personalized PD care. However, significant variability in electrophysiology, both within and between patients, must be taken into account when developing personalized treatments. There are many sources of variability, including, but not limited to the heterogeneous nature of PD, the expansive DBS stimulation parameter space and the effect of various medications. Modeling this variability will requires large and well-labeled data sets that link brain physiology to continuous objective metrics. These multimodal datasets can possibly be incorporated into routine clinical care to inform decisions about DBS – from patient selection, to DBS programming, to adaptive DBS (Gilron et al., 2021a,b; Fleming et al., 2022). However, collection and integration of these data require the development of new tools (Chen et al., 2021a). Using Rune Labs, we show an example of one patient who was continuously monitored for an entire year before and after implantation using a sensing enabled DBS, and we highlight ways in which this dataset paints a rich picture as compared to standard clinical scales. Notably, you can observe the variability in tremor fluctuations throughout the year with standard clinical tremor scores only capturing a small subset of this variability (Figure 6A). Both tremor and average STN beta were reduced as tremor was increased during routine clinical care (Figures 6B, C). Taken together, this highlights the role in which rich datasets pairing chronically recorded neurophysiology and objective data may be used to develop models that can inform stimulation or medication titration.

The DBS Think tank has encouraged global participation and in that spirit advances from Asia, Europe, and Australia were all covered.

## Updates from Europe

### Advancements in deep brain stimulation for psychiatric disorders

Deep brain stimulation (DBS) was introduced for the treatment of psychiatric disorders at the end of the 20th century, beginning with Gilles de la Tourette syndrome (GTS) and OCD. Since that time, the potential of this treatment has been further explored for other psychiatric indications in otherwise treatment resistant patients. A current meta-analysis conducted by Wehmeyer et al. (2021) summarized the results of studies with DBS in GTS and demonstrated that chronic DBS with different targets was associated with significant tic-reduction, with pallidal stimulation in this paper showing a possible advantage. Connectivity studies facilitated a more individualized approach for GTS patients with different symptoms and different comorbidities.

The effectiveness of DBS for OCD has been shown in several recent studies with different brain targets such as the medial dorsal nucleus of the thalamus, ventral anterior nucleus of the thalamus, medial forebrain bundle, subthalamic nucleus and inferior thalamic peduncle (Mallet et al., 2008; Maarouf et al., 2016; Lee et al., 2019). In a recent study from Li et al. (2020), a unified pathway between the dorsal anterior cingulate, ventrolateral prefrontal cortex and the anteriomedial STN was identified and was associated with a beneficial clinical outcome. These results could be replicated in further follow-up studies in different sets of patients. Several targets have also been described for treatment resistant depression. A recent study from Coenen et al. (2019) showed a long-term antidepressant effect of superolateral medial forebrain bundle DBS in a sham-controlled trial.

Deep brain stimulation (DBS) for patients with pathological aggressiveness and self-injurious behavior continues to be a controversial therapy option. A case-series from Torres et al. (2020) demonstrated a favorable long-term clinical outcome in the majority of patients with hypothalamic DBS. In summary DBS has proven to be an effective treatment option in several psychiatric disorders and there may be other indications emerging.

### Adaptive DBS for movement disorders–opportunities with externalized DBS hardware

The recent advent of sensing-enabled DBS has facilitated the recording of neural signals from chronically implanted electrodes during unconstrained activity and activity occurring in a naturalistic environment. Comparatively, perioperative subcortical sensing when the DBS electrodes are temporally externalized has been performed for more than 20 years. Despite the limitations of the methods on short recording time, constrained testing environment and potential stun effects, working with externalized patients offers unique advantages for research including: excellent signal to noise ratio, high sampling rate, and the possibility of accurately synchronized recording of other signals such as EEG, MEG, and EMG. These methods could offer new insights on the underlying circuit pathophysiology, and how cortical and subcortical neural oscillations could translate into muscle activities in behavior. It also could offer unique opportunities to test new algorithms and hardware, without being limited by what is feasible with an existing implantable device.

There are a few projects which demonstrate how we are taking advantage of this research opportunity to: (1) better understand the role of STN in gait control and to drive forward adaptive DBS for gait difficulties; (2) to explore the use of a machine learning based approach to detect specific brain states to drive closed-loop DBS for essential tremor; (3) to test and to compare different signal processing and control algorithms for adaptive DBS for PD while using beta amplitude as the feedback signal; and (4) to design and to test a new translational neuroscience research tool with improved performance on sensing during the actual stimulation.

### Deep brain stimulation for non-motor symptoms in Parkinson’s disease

Parkinson’s disease is associated with a multitude of non-motor symptoms (NMS) throughout the course of disease (Chaudhuri and Schapira, 2009). Sleep, autonomous functions, urogenital control, sensory perception and in particular mood and cognitive function can be impaired and have a profound impact on quality of life. Although often overshadowed by the visible motor fluctuations, NMS motor symptom severity also fluctuates with brain dopamine levels and can improve with continuous dopaminergic stimulation. This indicates that at least some NMS may result from hypodopaminergic brain circuit dysfunctions, while others are thought to reflect neurodegenerative loss of function. Deep brain stimulation has been used to explore the pathophysiology of several NMS in a systematic approach by comparing the effect of a levodopa challenge to a targeted intervention in the basal ganglia loop on non-motor readouts (Dafsari et al., 2018; Petry-Schmelzer et al., 2019; Reich et al., 2022). STN-DBS was shown to have direct impact on a central brain circuit regulating urinary bladder sensation and thereby reducing urinary urgency in PD (Herzog et al., 2006). Likewise emotional perception, drive and mood were elevated by STN-DBS in acute stimulation experiments to a similar extent as during an acute levodopa challenge. Neuroimaging and connectomic studies indicate that the optimal stimulation site for NMS within the subthalamic nucleus may differ slightly from the motor sweetspot of STN-DBS, but better tools for objectively and reliably measuring NMS may be required for mapping the NMS effects of DBS (Petry-Schmelzer et al., 2019).

Impulse control disorders, hypomania, compulsive levodopa intake and hyperactivity in advanced PD have been ascribed to dopaminergic sensitization following a similar mechanism as dyskinesia induction by pulsatile dopamine replacement therapy. These hyperdopaminergic NMS may also indirectly benefit from subthalamic DBS due to reduced medication requirements (Lhommée et al., 2012). First clinical evidence has demonstrated a favorable impact of STN-DBS on hyperdopaminergic behavioral symptoms in PD, which evolves over several months in parallel with dopaminergic drug withdrawal. In summary, non-motor does not mean non-treatable and NMS burden should therefore be evaluated and stratified for a potential therapeutic impact during the selection process for DBS surgery.

## Updates from Asia

### Insights from modulation of intracranial recordings on cognitive processes

Recent studies by Dr. Voon and colleagues focus on stimulation-sensitive biomarkers by investigating local field potential physiology in the context of cognitive processes and its sensitivity to time-locked stimulation. In her discussion, Dr. Voon highlights studies focusing on the STN in PD. First, she showed that the physiology underlying objective markers of risk can be dissociated from subjective betting (Manssuer et al., 2022). High frequency acute STN stimulation decreased the risk taking possibly through modulating STN theta frequency. STN DBS was associated with increased impulsivity with hastened “responding under conflict.” These findings emphasize the heterogeneity of impulsivity with potential implications for disorders of addiction. Second, she showed the capacity to enhance subjective positive emotional bias through targeting the late alpha desynchronization to affective stimuli by using alpha-specific frequency acute STN stimulation which enhanced alpha power. Patients with depressive symptoms appeared to have a greater positive bias to alpha frequency rather than high frequency stimulation, again highlighting the potential impact on comorbid depression. Further updates from ongoing randomized controlled trial studies in China include: major depression targeting the bed nucleus of the stria terminalis demonstrating changes in low frequency oscillatory activity with improvements in depression; multicenter studies targeting nucleus accumbens and ventral internal capsule for opioid use disorder and obsessive compulsive disorder.

### DBS in China, from clinical to clinical research

Closed-loop neuromodulation is an inevitable trend of the expansion of DBS and naturally, it is also a research hotspot. Closed-loop DBS does not use additional electrodes to collect brain data, but relies on the stimulation electrodes. Closed-loop DBS could synchronously record LFPs of the target brain area during stimulation, especially recording long-term outcomes (Qian et al., 2016). Therefore, closed-loop DBS will become more important and could shed light on the chronic DBS mechanism. To accurately describe the information from the deep brain target, it will be necessary to remove the artifacts, for which a lot of preliminary preparations have been made (Qian et al., 2017a,b; Chen et al., 2021b,c). Based on 6 months recordings of STN-LFP signal, Dr. Li and colleagues recently reported the potential characteristics of chronic neuromodulation effects in the stimulated target (Chen Y. et al., 2020). By making chronic synchronous recording possible, closed-loop DBS could be regarded as a fully-implantable brain-machine interface (BMI). Recently, the Li lab investigated the performance of using a closed-loop neurostimulator as a motor BMI (Chen J. C. et al., 2022). By decoding movement from STN-LFP information, the system achieved a typical two-dimensional center-out task to simulate virtual wheelchair control.

In 2020, Shen et al. (2020) reported the first systematic work of studying the DBS regulatory mechanism based on 3T magnetic resonance compatibility technology (Jiang et al., 2014; Bai et al., 2016; Zhang et al., 2019). The authors conducted follow-up studies after 1, 3, 6, and 12 months of implantation of bilateral STN in 14 PD patients. With a block-design that interleaved stimulation on and off while performing fMRI, the brain function states were analyzed with high repeatability and reliability. The authors observed that STN-DBS could regulate the cerebellum. Two distinct circuits showed different frequency and time-dependent modulatory effects. The circuit involving the globus pallidus internus (GPi), thalamus, and deep cerebellar nuclei was sensitive to stimulation frequency and was more activated under high-frequency. The circuit involving the primary motor cortex (M1), putamen, and cerebellum was deactivated and remained unchanged under different frequencies. In contrast, deactivation in the M1 circuit was gradually enhanced over time, however the GPi circuit revealed no change.

### Role for the amygdala in treatment-refractory obsessive-compulsive disorder using deep brain stimulation

The neural basis for OCD, a disabling psychiatric condition with a lifetime prevalence of 2–3%, is uncertain (Gadot et al., 2022). The classical neurobiological models of OCD based primarily on dysfunctional parallel cortico-striatal loops have been questioned, while the precise role of other implicated brain regions, such as amygdala and cerebellum, also remains unclear (Kammen et al., 2022). Here the Queensland Brain Institute report single unit recordings from the BNST region during DBS implantation which showed that action potentials were broader in patients with more severe OCD. Functional neuroimaging data collected before electrode implantation showed enhanced amygdala and cerebellar responses to negative emotional pictures in OCD patients as compared to healthy controls. Cerebellar vermis responses to negative pictures explained 97.2% of inter-subject symptom variance in OCD patients. Functional connectivity between amygdala and cerebellar vermis predicted 95% of inter-subject variance in OCD symptoms following 23 weeks of DBS therapy applied to the bed nucleus of the stria terminalis within the extended amygdala. These results indicated a crucial role for amygdala-cerebellar functional connectivity in mediating OCD symptomatology and the therapeutic effect of DBS.

The DBS think tank deliberately and proactively addresses neuroethical issues and dilemmas facing the field. Emerging issues included measuring what matters, informed consent and in addressing legal issues in psychiatric neuromodulation.

## Neuroethics cases: Dilemmas that inform the future of neuromodulation

### Measuring what matters

Empirical data can be used to demonstrate that commonly used patient outcome measures do not assess patients’ primary goals for pursuing DBS for PD. Similarly, existing personality measures do not fully capture the public understandings of personality. Our empirical data highlight that DBS used to treat the motor symptoms of PD significantly helps patients achieve their behavioral goals and may be restorative to the patients’ most valued personality characteristics. Additionally, their ratings following DBS could possibly be “closer” to retrospective ratings reflecting their personality prior to disease onset.

These observations highlight the importance of soliciting a patients’ perspective and their lived experiences in order to develop better outcome measures. Outcome measures that are more relevant to patients’ perspectives, values, and goals are directly related to best informed consent practices. Our recommendations extend to neuromodulation trials for other indications, particularly neuropsychiatric trials in which the behavioral goals and personality factors may be complex and nuanced. We encourage other clinical research teams to systematically assess patients’ goals and values and to incorporate outcome measures better reflecting those values. We highlight the need to include the voices of under-represented minorities who have been historically ignored in an effort to ensure that we do not inadvertently perpetuate healthcare inequities and assume that the data collected (primarily from white participants) reflect other groups values and goals. Partnerships with humanities scholars will be essential for expanding the promotion of more patient centered, inclusive healthcare within neuromodulation.

### Ethical issues in intraoperative neuroscience research: Assessing recall of informed consent and motivations for participation

The use of neurosurgical patients as human research subjects raises important ethical considerations, yet a thorough empirical examination of these issues in a participant population has been lacking. The Wexler lab aimed to empirically investigate the ethical concerns regarding informed consent and voluntariness in PD patients undergoing DBS participating in an intraoperative neuroscience study. Two semi-structured 30-min interviews were conducted preoperatively and postoperatively *via* telephone. Interviews assessed a subjects’ motivations for participation in the parent intraoperative study, recall of information presented during the informed consent process, and postoperative reflections on the research study. Twenty-two participants completed preoperative interviews and twenty participants completed postoperative interviews. All participants cited altruism or advancing medical science as “very important” or “important” in their decision to participate in the study. Only 22.7% (*n* = 5) correctly recalled one of the two risks of the study. Correct recall of other aspects of the informed consent was poor (36.4% for study purpose; 50.0% for study protocol; 36.4% for study benefits). All correctly understood that the study would not confer a direct therapeutic benefit to them. Though research coordinators were properly trained and the informed consent was administered according to protocol, participants demonstrated poor retention of study information. While intraoperative studies aimed to advance neuroscience knowledge may represent a unique opportunity to gain fundamental scientific knowledge, improved standards for the informed consent process can help to facilitate ethical implementation.

### Psychiatric neurosurgery laws and incapable patients: What would a model law say?

Mental health legislation in multiple countries, specifically addressing neurosurgery for psychiatric disorders, includes psychiatric applications of DBS within the scope of the laws (Nadler and Chandler, 2019; Chandler et al., 2021). Many of these laws are quite recent (Mental Health Act, Scotland, 2003; Mental Health Act, Australia, 2014; Mental Healthcare Act, India, 2017), revealing continued social and legal attention to neurosurgical treatments especially to address psychiatric conditions. These laws often address the following general issues: restricted eligibility of particular populations for psychiatric neurosurgery (particularly incapable patients, children, prisoners and involuntarily hospitalized patients), independent pre-surgical approval processes sometimes including courts or mental health tribunals, and record-keeping and post-surgical reporting requirements (Chandler et al., 2021). The 1977 report on psychosurgery by a US National Commission made recommendations in each of these areas. The report also suggested that it would be unfair to categorically exclude certain groups of patients from access, despite concerns about the use of invasive neurosurgery (United States National Commission for the Protection of Human Subjects of Biomedical and Behavioral Research, 1977). If and when evidence builds about the efficacy of DBS for psychiatric purposes, this question may become more pressing for jurisdictions that currently exclude particular populations from access, in order to protect them from harm. A critical part of the path forward will be ensuring that the views of people who have severe mental health challenges will be included as central in the discussion about the legal regulation of this field. A further legal question of importance will be how mental health care funding parity and anti-discrimination laws in countries with various health care funding models might influence the underfunding of all mental health care; perhaps including DBS for psychiatric applications.

## DBS candidacy: The next frontier in emerging therapies

An important area in neuromodulatory therapies has been identification of candidate biomarkers, use of AI technology and building wire diagrams for translational neuroscience. The DBS Think Tank addressed the emerging issues in the DBS field.

### Identification of candidate neural biomarkers of obsessive-compulsive symptom intensity in ecologically valid environments

Despite the success of DBS for the treatment of refractory OCD, there are currently no robust neural signatures for obsessive-compulsive (OC) symptoms. This shortcoming may be due to limited opportunities available for conducting intracranial electrophysiological recordings in natural environments where fluctuations in symptoms may occur. Recently available DBS platforms offer a way past this hurdle, allowing for streaming of intracranial neural activity both at home and in the clinic. Here, our goal was to identify neural correlates of OC symptom intensity. Provenza et al. conducted longitudinal intracranial recordings in five participants with refractory OCD implanted with recording-capable DBS devices targeted to the ventral capsule/ventral striatum (VC/VS) or bed nucleus stria terminalis (BNST). Provenza and colleagues captured LFPs at home during naturalistic exposures to OCD triggers. All five participants who completed the study were clinical responders to DBS therapy. Using the intracranial data collected during OCD exposures, the team computed correlations between spectral power and OCD symptom severity. They then identified low delta-band power as a candidate neural biomarker of OC symptom intensity during symptom provocations in one participant (left VC/VS: *R* = −0.59, *p* = 0.01; right VC/VS: *R* = −0.56, *p* = 0.04) (Provenza et al., 2021). This signal has potential utility for classification of symptom intensity in adaptive DBS systems for OCD. Continued opportunities for long-term, naturalistic intracranial electrophysiological recordings will help to propel biomarker discovery for OCD and other psychiatric disorders.

### Building a wiring diagram of the brain: Tools for translational neuroscience

The efficacy of DBS is highly dependent on targeting the “right” connections. Mapping anatomical connectivity of the human brain is not a straightforward task, particularly when it comes to white matter organization. The Heilbronner lab has worked on the methodologies used to uncover anatomical connectivity. Diffusion tractography, for example, is applicable in humans and can be used to interrogate the connectivity of the whole brain, but it frequently fails to generate accurate fiber trajectories. Anatomical tract-tracing, by contrast, is highly accurate, but has been limited to use in non-human animal models and is fundamentally not whole-brain. Dr. Heilbronner highlighted how deliberately cross-species and cross-modal pipelines can help us to achieve more accurate wiring diagrams of the human brain as a method to aid in neuromodulation. These diagrams can provide neuroanatomical underpinnings of complex behaviors and resting-state fMRI results.

### A novel, simple, rapid, and inexpensive biomarker of the ventral reward system

**FIGURE 7:**
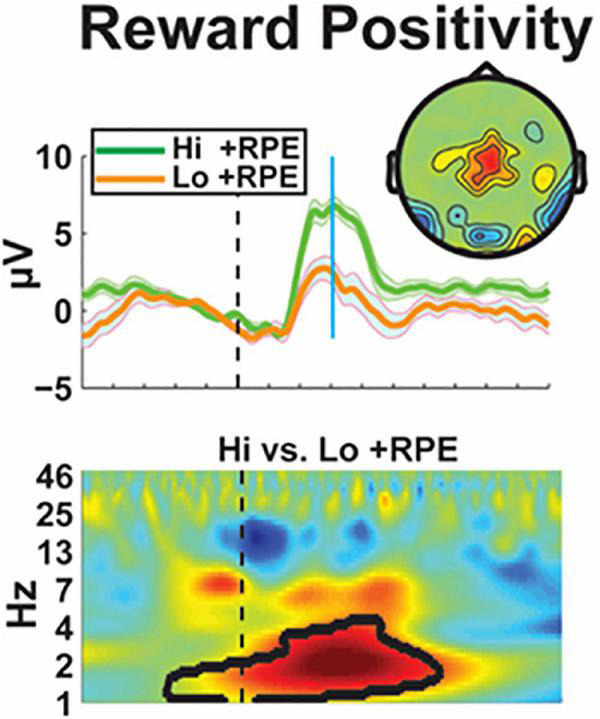
Electrophysiological characterization of reward positivity–An electrophysiological biomarker of the ventral reward system is demonstrated by the positive reward prediction error (+ RPE). This signal can be seen when receiving a “highly surprising reward” (Hi + RPE) or an “expected reward” (Lo + RPE) after a task. This differential effect can then be used to identify electrophysiologic associations with the reinforcement learning test paradigm to characterize reward value.

Electrophysiology (EEG) is a direct measure of neuronal processes, and it is uniquely sensitive to canonical neural operations that underlie emergent psychological operations. These qualities make EEG well-suited for the identification of aberrant neural mechanisms that underlie complicated disease states. The Cavanagh lab reviewed the qualities of a novel biomarker of the ventral reward system: the event-related potential component known as the Reward Positivity (RewP). The RewP emerges as an over the cortical midline as a positive polarity deflection from ∼200–500 ms following reward receipt. The RewP is not only specifically elicited by rewards, but it is also sensitive to the major computational construct used to define reward value: RewP amplitude scales with the degree of the positive reward prediction error. They presented the magnetoencephalographic source estimation that the RewP is generated by ventromedial prefrontal cortex (Figure 7). Moreover, they showed that the diminished RewP in major depression is likely due to hypoactivity in these areas, including subgenual cingulate. Translational applications of the RewP will be presented, including a novel mouse model which will facilitate bench-to-bedside applications. The RewP will be contrasted with an established mechanistic biomarker of cognitive control: frontal theta band activity, which is reliably enhanced in anxiety disorders. Together, these findings will motivate the use of EEG biomarkers, including frontal theta and the RewP, for assessing the efficacy of psychiatric deep brain stimulation on canonical neural circuits.

## What’s next for clinical neuromodulation

An important aspect of neuromodulatory therapy is asking the question what’s next? New approaches for dystonia DBS have been moving into clinical practice. The use of AI is getting closer to informing clinical decision making. We discussed the idea that there may be a return to “brain lesioning,” and how the field is moving closer to true brain-computer interfaces.

### Cerebellar neuromodulation for acquired dystonia

Dystonia in the setting of cerebral palsy (CP) is the most common cause of acquired dystonia in childhood, and its management can be challenging. DBS of basal ganglia or thalamus has played a major role in the treatment of isolated dystonias, however its efficacy in dystonic CP is lower. This may be due to underlying structural damage, lack of improvement of comorbid choreoathetosis and spasticity, and an increased risk of hardware complications.

The cerebellum may represent an alternative brain target for dystonic CP. It has a recognized role in dystonia pathophysiology; it is frequently spared from hypoxic ischemic damage, and small studies have shown the promise of cerebellar stimulation in improving spasticity and CP-related movement disorders.

Dr. San Luciano presented preliminary data from three dystonic CP participants with bilateral cerebellar DBS targeting the dorsal (microgyric, motor) dentate nucleus using Medtronic Percept. Cerebellar LFPs were recorded and a prominent alpha rhythm (∼10 Hz) was identified, which decreased in amplitude and variance at higher stimulation amplitudes, a possible physiomarker of dystonia. Beta band peaks (∼20 Hz) were also present. All participants experienced subjective variable improvements in symptoms, including in hand coordination, head control, speech clarity and fluency and perceived limb tightness, collectively representing ∼20–40% rating scale improvement.

Dr. San Luciano and colleagues proposed a larger study of cerebellar DBS in children and young adults with dystonic CP. They will characterize cerebellar LFPs related to clinical assessments, wearable monitors, and relation to stimulation; perform pre and post- postoperative volumetric structural and functional MRI and diffusion tensor imaging to identify candidate imaging markers of baseline disease severity and response to DBS, and to test its efficacy for improving quality of life, clinical assessments and objective kinematic metrics in an N-of-1 clinical trial design.

### Informing clinical decisions in psychiatric neuromodulation with AI

When pursuing new DBS indications, we must establish clinical guidance in each step of the process, including patient selection, DBS targeting, and ongoing therapeutic decision making. This “decision making step” is especially challenging in depression, where common assessment methods are often based on surveys with known biases and that can reflect non-specific symptoms. As a specific example, depression DBS patients often experience instability in measures such as the Hamilton Depression Rating Scale (HDRS), and clinical teams must determine if the HDRS increases reflect worsening depression (indicating dose adjustment) or transient mood disturbances not warranting intervention. Dr. Rozell presented their ongoing work using longitudinal LFP recordings from subcollosal cingulate DBS patients and novel explainable AI techniques to develop objective biomarkers of stable recovery. In addition to showing generalization of the derived biomarkers across multiple patient cohorts (with different clinical teams and implantable devices), they showed a series of case studies illustrating how this biomarker could support clinical decision making during DBS therapy. These case studies included responding patients with HDRS increases due to transient anxiety, and scenarios where relapses (reflected in HDRS scores) were significantly preceded by biomarker changes that could have indicated the need for therapeutic intervention.

### Is the future of DBS a return to brain lesions?

Historically, brain lesions provided the foundation for localization of symptoms and were used as a treatment for brain diseases. One of James Parkinson’s original 6 patients experienced relief of tremor following a focal stroke. This serendipitous observation motivated neurosurgical interventions, eventually leading to DBS. While the past of DBS has been based on lesions, many assume that the future of DBS is based on electrophysiology. Significant effort is being put into identifying electophysiological biomarkers and targeting these biomarkers with adaptive or closed loop stimulation, and great progress has been made. However, an alternative (and controversial) possibility is that the future of DBS is actually a “return to brain lesions.” Recent advances have improved our ability to map lesion-induced effects to specific brain circuits, including lesions that provide relief of symptoms such as tremor or addiction. These lesion-based localizations align with DBS benefits and side effects such as memory decline and depression. Improved localization can lead to more precise neuroanatomical treatment targets, which may mitigate the traditional advantages of DBS over lesions such as “reversibility and tunability.” New tools for creating brain lesions such as high intensity focused ultrasound could facilitate lesions to be placed without a skin incision and are already in clinical use for tremor. Although there are limitations and caution is warranted with irreversible lesion-based interventions, improved precision may 1 day make lesions preferable over DBS. In summary, improvements in lesion-based localization are refining our therapeutic targets and improved technology have been providing new ways to create lesions, which together may lead to a change in the relative value of DBS versus lesions.

## Emerging consumer BCI

Controlling computers with human brain signals is quickly becoming a reality through brain-computer interfaces (BCI). Development of BCI devices has been based on academic research that has largely contributed to our understanding of brain functions. Over the past decade many laboratories have dedicated their research to improving our knowledge on how neurons in the brain encode movements, decision, and behavior. Dr. Henderson’s lab (among others) investigated movement-related signals in human motor cortex to advance neural prosthetics, including translation of a high-performance neural prosthesis (Gilja et al., 2015), typing at ∼8 words per minute using a brain-controlled cursor (Pandarinath et al., 2017), control of a tablet computer using neural signals (Nuyujukian et al., 2018), and high-performance brain-to-text communication using decoded handwriting (Willett et al., 2021). These innovations have helped to demonstrate the potential practical uses for an interface between the brain and a computer system.

Today, many companies have integrated this BCI technology into their roadmap. Neuralink, led by CEO Elon Musk, is one of the most advanced BCI companies in the field and has been developing a fully-implantable, wireless, high-channel-count device and an automated robotic system for reliable and efficient implantation (Musk and Neuralink, 2019). This system, called the link, records neuronal activity, decodes the information through AI algorithms and sends a command back to the computer. It has been successfully implanted and tested in non-human primates and is nearing clinical trials to provide assistance for people with neurological disorders. Other companies using different technology to record and stimulate the brain are also emerging. Motif Neurotech is a new company that aims to stimulate the brain through magnetoelectrics, a process by which oscillating magnetic fields are converted to oscillating electric fields (Chen J. C. et al., 2022). This technology uses a miniature neurostimulator the size of a pea that can be delivered with minimally invasive surgical techniques and will stimulate neural tissue without the need for wires or batteries. An external power source, housed in a wearable, will provide the energy and stimulation pattern. To maximally leverage the advantages of this technology, the initial applications will focus on disorders not requiring continuous stimulation, as compliance with use of the wearable will be challenging. Disorders that can be successfully treated with episodic therapy such as transcranial magnetic stimulation (TMS) may be ideal targets. Individuals with psychiatric disorders, for example, often respond to TMS therapy, but relapse rates are high. The ultimate neurostimulation strategy will be designed to allow individuals to deliver therapy from home, using a convenient and effective dosing plan.

Clinically viable BCI will likely be soon available to help people with medical conditions. However, BCI is emerging as the next “trendy” technology and expending beyond the medical application. There is consensus that consumer BCI will soon be part of everyday life. Although we should not be scared of this BCI emergence, it is important to understand these technologies and to assess the risks associated. These risks and issues will inform effective policies to protect users. Data privacy and sharing, full disclosure on the system capability and limitations, responsibility of the company beyond clinical trials will all be important risks. These risks should be debated and proactively addressed with industry as an equal partner.

## Conclusion

**FIGURE 8:**
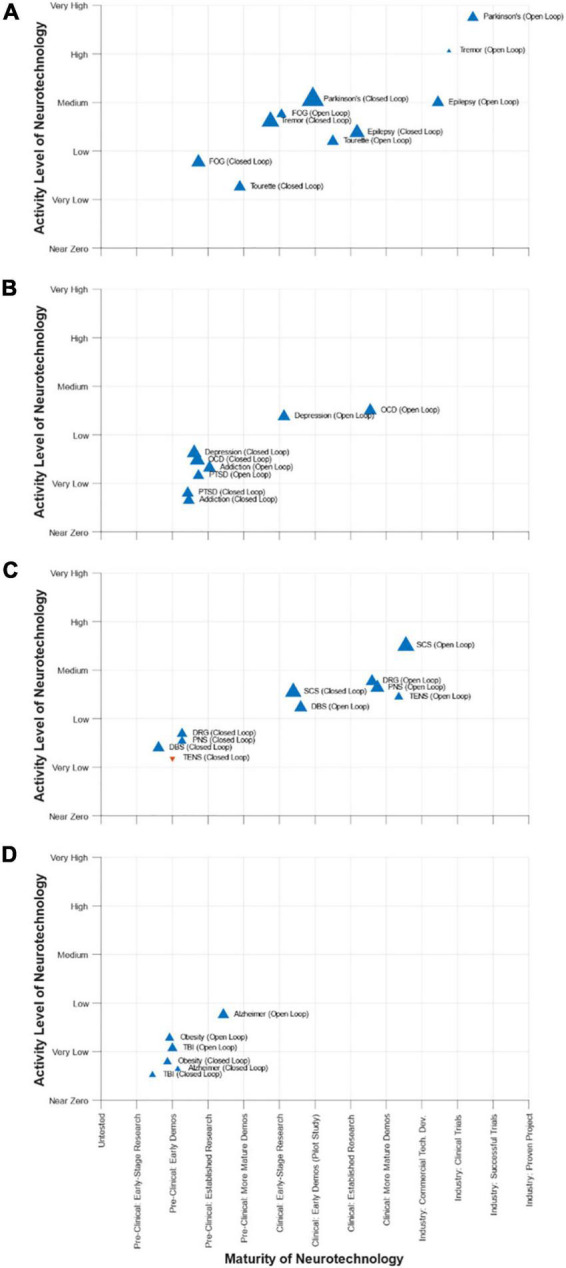
DBS-Think-Tank Neurotechnology Activity-Maturity Graphs–This figure presents four graphs that illustrate the perceptions of DBS-Think-Tank attendees about the maturity, activity, and change in activity of a variety of neurotechnologies. The neurotechnologies are organized into the following four groups and graphed separately: **(A)** movement disorders, **(B)** psychiatric disorders, **(C)** pain disorders, and **(D)** other syndromes. The upward pointing blue triangles represent increasing activity and downward pointing orange triangles represent decreasing activity. The magnitude of the rate of change is proportional to the size of the triangles. The definitions of the abbreviations used to identify each triangle are as follows: DBS, deep brain stimulation; FOG, freezing of gait; OCD, obsessive-compulsive disorder; PTSD, posttraumatic stress disorder; PNS, peripheral nerve stimulation; TENS, transcutaneous electrical nerve stimulation; DRG, dorsal root ganglia stimulation; SCS, spinal cord stimulation; TBI, traumatic brain injury. The data presented were derived from a survey that had a total 45 respondents. Characteristics of the respondents were as follows: affiliation (75% academic, 20% industry, 5% government), background (89% clinical research, 57% technology development, 52% engineering, 45% provider of clinical care, 23% biological science, 14% technology commercialization, 11% fund research and/or development, 11% physical science, 7% entrepreneurship, 5% regulation, 5% reimbursement, 5% management/administration, and 2% patient), and years of experience (18% with 0 to 5 years, 25% with 5 to 10 years, 23% with 10 to 15 years, 18% with 15 to 20 years, 11% with 20 to 25 years, and 5% with 25 to 30 years).

After the conference, the DBS Think Tank participants completed a survey that captured their views about the maturity, activity level, and rate of growth (or reduction) in activity for the use of neurotechnology to address selected movement disorders, psychiatric disorders, pain, and other conditions. The results from the survey are summarized in Figure 8. A key feature of the survey was the assessment of open-loop and closed-loop neuromodulation therapies for each condition. Although the maturity of closed-loop neuromodulation is nascent to non-existent for some indications, it is showing great promise for others. It will be interesting to see the creation, evolution, and growth of closed-loop neuromodulation in the years ahead and the ability of the DBS Think Tank community to anticipate it.

This year, the DBS Think Tank X sessions were all led and organized by women leaders in neuromodulation. The DBS Think Tank X traced the milestones in depression DBS and we discussed the re-introduction of a major clinical trial in this area. The Think Tank X addressed both the challenges and opportunities for adaptive or for closed loop DBS. There was discussion as to whether the closed loop approach would be optimal for “all patient groups” or will be more appropriate for select groups of individuals with specific symptom profiles. The DBS Think Tank X discussed the emergence of new targets, electrical biomarkers, AI and challenges in neuroethics especially as we move closer to true brain-computer interfaces. It was clear from the 3 days of discussion that many groups are using animal models to drive the science along with true intra-operative research approaches. The neuromodulation field continues to rapidly grow with an estimated 244,000 implants worldwide.

## Data availability statement

The original contributions presented in this study are included in the article/supplementary material, further inquiries can be directed to the corresponding author.

## Ethics statement

The studies involving human participants were reviewed and approved by the individual academic institutions. All participants provided their written informed consent prior to participation in the studies. The animal study was reviewed and approved by individual institutional animal welfare committees at the respective academic institutions.

## Author contributions

All authors listed have made a substantial, direct, and intellectual contribution to the work, and approved it for publication.
